# Recruitment and Ongoing Engagement in a UK Smartphone Study Examining the Association Between Weather and Pain: Cohort Study

**DOI:** 10.2196/mhealth.8162

**Published:** 2017-11-01

**Authors:** Katie L Druce, John McBeth, Sabine N van der Veer, David A Selby, Bertie Vidgen, Konstantinos Georgatzis, Bruce Hellman, Rashmi Lakshminarayana, Afiqul Chowdhury, David M Schultz, Caroline Sanders, Jamie C Sergeant, William G Dixon

**Affiliations:** ^1^ Arthritis Research UK Centre for Epidemiology University of Manchester Manchester United Kingdom; ^2^ NIHR Manchester Musculoskeletal Biomedical Research Unit Central Manchester University Hospitals NHS Foundation Trust Manchester United Kingdom; ^3^ Department of Statistics University of Warwick Coventry United Kingdom; ^4^ Oxford Internet Institute University of Oxford Oxford United Kingdom; ^5^ School of Informatics University of Edinburgh Edinburgh United Kingdom; ^6^ uMotif London United Kingdom; ^7^ Centre for Atmospheric Science, School of Earth and Environmental Sciences University of Manchester Manchester United Kingdom; ^8^ Medical Sociology, Division of Population Health, Health Services Research and Primary Care University of Manchester Manchester United Kingdom

**Keywords:** epidemiology, mHealth, chronic pain, methods

## Abstract

**Background:**

The huge increase in smartphone use heralds an enormous opportunity for epidemiology research, but there is limited evidence regarding long-term engagement and attrition in mobile health (mHealth) studies.

**Objective:**

The objective of this study was to examine how representative the Cloudy with a Chance of Pain study population is of wider chronic-pain populations and to explore patterns of engagement among participants during the first 6 months of the study.

**Methods:**

Participants in the United Kingdom who had chronic pain (≥3 months) and enrolled between January 20, 2016 and January 29, 2016 were eligible if they were aged ≥17 years and used the study app to report any of 10 pain-related symptoms during the study period. Participant characteristics were compared with data from the Health Survey for England (HSE) 2011. Distinct clusters of engagement over time were determined using first-order hidden Markov models, and participant characteristics were compared between the clusters.

**Results:**

Compared with the data from the HSE, our sample comprised a higher proportion of women (80.51%, 5129/6370 vs 55.61%, 4782/8599) and fewer persons at the extremes of age (16-34 and 75+). Four clusters of engagement were identified: high (13.60%, 865/6370), moderate (21.76%, 1384/6370), low (39.35%, 2503/6370), and tourists (25.44%, 1618/6370), between which median days of data entry ranged from 1 (interquartile range; IQR: 1-1; tourist) to 149 (124-163; high). Those in the high-engagement cluster were typically older, whereas those in the tourist cluster were mostly male. Few other differences distinguished the clusters.

**Conclusions:**

Cloudy with a Chance of Pain demonstrates a rapid and successful recruitment of a large, representative, and engaged sample of people with chronic pain and provides strong evidence to suggest that smartphones could provide a viable alternative to traditional data collection methods.

## Introduction

In the United Kingdom, 70% of adults own a smartphone, over half of whom use apps [1]. This growth in smartphone use within the general population heralds an enormous opportunity for epidemiology and population-health research [2-4], allowing data collection to be integrated into people’s lives. Smartphone apps for health monitoring can potentially deliver frequent and regular self-reported symptoms, whereas sensors on smartphones can aid collection of new data types, including position, movement, and environmental exposures [5].

Despite high expectations about mobile health (or mHealth) [4] studies and initial evidence that mHealth studies can recruit at scale [5], limited evidence exists on representativeness of populations who participate in digital health studies and patterns of engagement over time [6-8]. This is particularly pertinent, given the known existence of both primary and secondary digital divides, in which younger adults from higher socioeconomic backgrounds are not only more likely to have access to a smartphone device but will also utilize them differently from older adults [1,9]. Thus, though younger adults are more likely to download apps and play games on their devices, older users primarily view their smartphone as a means of communication [1].

Although smartphones appear to offer a more rapid and mobile method of data collection without compromising completion rates obtained by traditional methods [10,11], relatively little detailed information is available regarding participant recruitment and retention, or engagement, in smartphone studies, particularly when compared with other traditional methods [12] or Web-based studies [13]. Engagement has previously been defined in ways which fail to account for the potentially variable patterns of use through time, including continuity of data entry [5,14-16], and this nonuniformity in definitions makes it difficult to draw conclusions regarding the viability of mHealth studies for longitudinal research.

Cloudy with a Chance of Pain is a UK smartphone-based, prospective cohort study investigating the link between the weather and pain in people with chronic pain. Specifically, Cloudy with a Chance of Pain seeks to investigate whether self-reported pain severity is associated with weather variables and whether the observed relationships differ between specific patient groups. Earlier research on this topic has been inconclusive [17], despite more than two-thirds of patients with musculoskeletal pain believing that there is an association between the weather and pain [18,19]. The numerous methodological challenges that have traditionally contributed to this ambiguity include small sample sizes, a lack of temporally rich data, and poor availability of data pertaining to geographical and meteorological variability. However, smartphone apps have the capacity to overcome these challenges, if they can recruit and continue to engage a representative study population.

The two aims of this paper were to examine how representative the Cloudy with a Chance of Pain study population is of wider chronic-pain populations and to explore patterns of engagement among participants during the first 6 months of the study.

## Methods

From January 20, 2016 to January 20, 2017, Cloudy with a Chance of Pain aimed to recruit over 1000 UK residents aged 17 or over who owned an Android or iPhone operating system (iOS; Apple Inc) smartphone, and who experienced pain for at least the preceding 3 months. The study was advertised through national and regional television, radio and newspaper media, social media, and via charity and patient partner organizations (Multimedia Appendix 1). Further information for interested participants was available on the study website [20].

To enroll in the study, participants downloaded the uMotif app [21] on their smartphone from the Apple App Store or Google Play Store. After completion of digital consent, the app enabled participants to report their symptoms daily for 6 months, or longer if willing. In the background, the smartphone’s Global Positioning System (GPS) reported hourly location, allowing linkage to local weather data from the Met Office (the UK’s national weather service) and investigation of the association between weather and pain. More details on the app and data collection are provided below.

Participants included in this analysis were those recruited between January 20, 2016 and February 29, 2016, with patterns of engagement examined through to July 20, 2016, 6 months from the study launch date. Participants provided a year of birth through the consent process to confirm that they were 17 years of age or older. Not everyone who downloaded the app used it, so eligibility was further restricted to those who had reported their symptoms at least once between enrollment and July 20, 2016.

Ethical approval was obtained in December 2015 from the University of Manchester Research Ethics Committee 4 (ref: ethics/15522).

### Data

#### Baseline Data

The baseline questionnaire collected demographic data: sex, year of birth, and first half of participant’s postcode. Participants reported the site of pain (eg, head, face, knee) and were able to report pain at multiple sites or having pain all over the body. Participants were asked to record whether they had been diagnosed (by a doctor) with rheumatoid arthritis, ankylosing spondylitis or spondyloarthropathy, gout or other calcium-crystal arthritis (eg, pseudogout), arthritis (type not specified), fibromyalgia or chronic widespread pain, chronic headache, or neuropathic pain. A free-text entry box was provided for any diagnoses not otherwise listed. Due to a coding error, diagnoses of osteoarthritis (OA) were not collected for the first 9 weeks of data collection, after which it was included within the above list. A push notification was sent out on March 24, 2016 asking existing participants to indicate whether or not they had the condition. Responses were received from 1157 of 8267 (13.99%) of participants recruited by March 24, 2016. For this reason, prevalence rates of OA are not provided in this paper.

Participants reported their use of paracetamol, nonsteroidal anti-inflammatory drugs (NSAIDs), simple analgesics, weak opiates, strong opiates, and drugs for neuropathic pain. Participants reported their use of glucocorticoids (steroids), synthetic disease modifying antirheumatic drugs (DMARDs), and biologic DMARDS. Participants could also report the use of other medications. If “other” was selected, a free-text entry box was provided.

**Figure 1:**
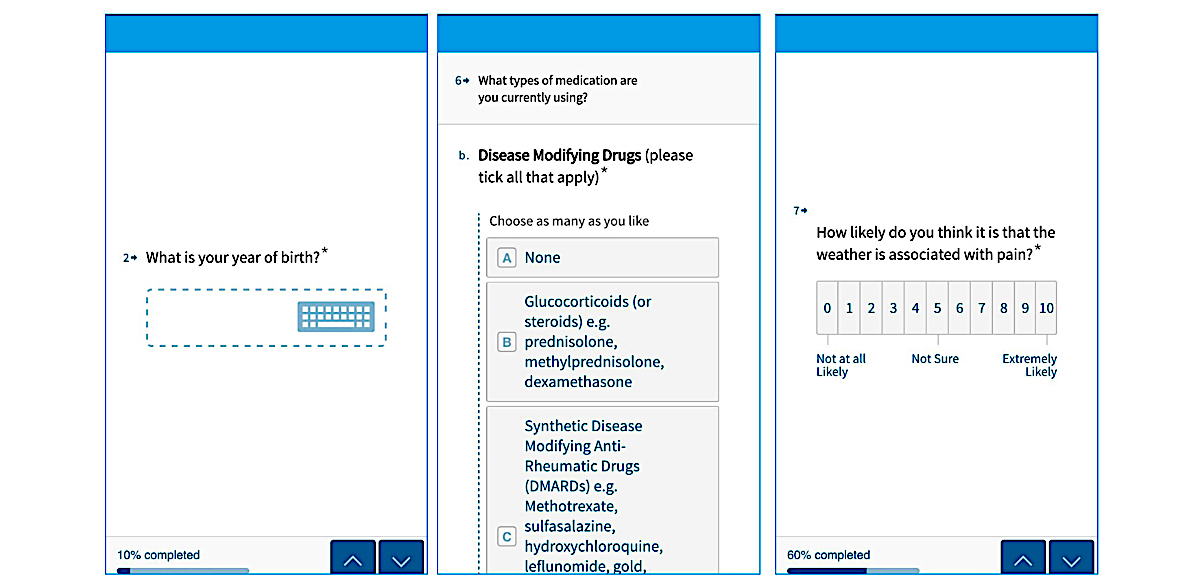
Screenshot of example baseline data collection.

Participants reported how likely they thought it was that the weather was associated with pain using a 0 to 10 numerical rating scale (NRS), where 0 indicated not at all likely and 10 indicated extremely likely. Participants were also asked which weather conditions they most felt were associated with pain, selecting from damp/rain, cold, heat, change in barometric pressure, change in temperature, and other (free-text box provided to specify belief). Examples of data-entry screens are shown in Figure 1.

#### Daily Symptom Domains

**Figure 2:**
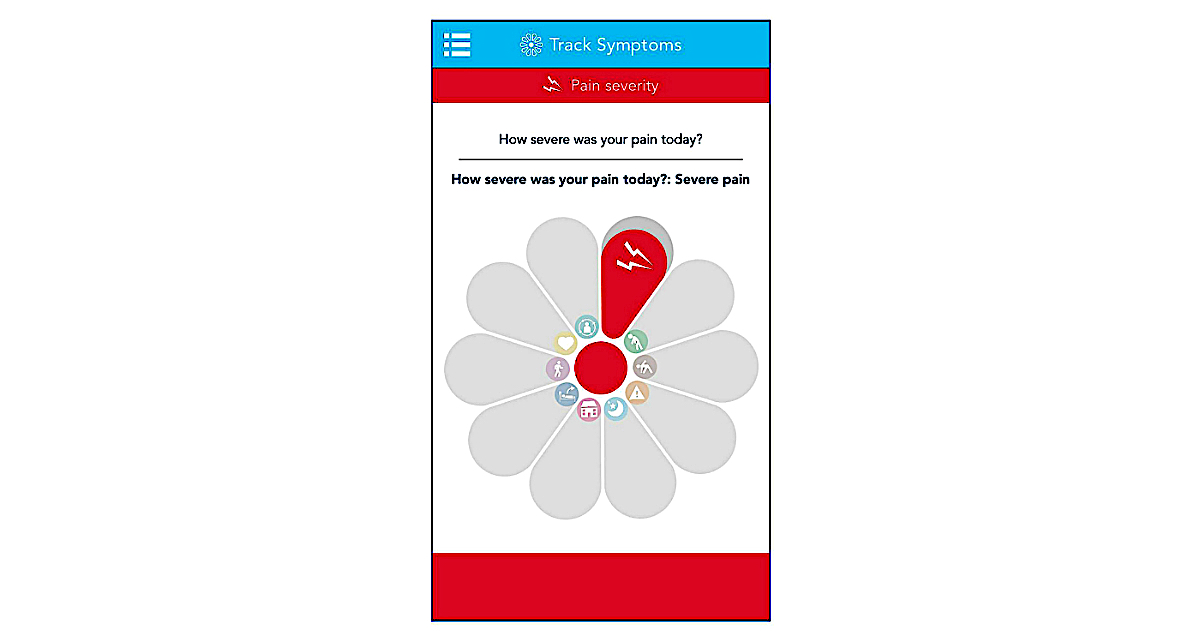
Screenshot of motif for daily symptom collection.

Following completion of the baseline questionnaire, participants were asked to report 10 symptoms every day using the uMotif app (Figure 2), prompted by a daily notification at 6:24 p.m. Each symptom was scored in five ordinal categories (eg, pain was scored as no pain, mild, moderate, severe, or very severe). The symptoms were pain severity, fatigue, morning stiffness, the impact of pain on activities, sleep quality, time spent outside, feeling tired on waking, physical activity, mood, and well-being. A study motif was considered complete when all 10 variables were reported at a single time point. The app was codesigned with a patient and public involvement group and refined after a feasibility study of 20 participants with rheumatoid arthritis [22].

### Analysis

#### Representativeness of Participants

To explore the representativeness of participants recruited to this study, we compared the age and sex distribution of participants with that of a sample of persons with chronic pain (≥3 months) from the Health Survey for England (2011) [23]. The Health Survey for England is a large-scale annual survey that has been conducted since 1994 and recruits a stratified random probability sample of private households within England. Full description of the methods of data collection are available elsewhere [24].

#### Engagement

We sought to define common patterns of engagement (ie, data entry), using a three-step process.

Following recruitment, individuals were labeled as engaged if they reported any of the ten symptoms on a given day. A first-order hidden Markov model [25,26] was then used to estimate the levels of engagement of participants, using the depmixS4 R package (I Visser, Netherlands)[27] (Multimedia Appendix 2). The model assumed three latent engagement states: high, low, and disengaged. The model was initialized assuming every participant started highly engaged. Furthermore, the model assumed that disengagement was an “absorbing state,” so that participants entering this state could not reengage with the study. Finally, clusters were defined according to different probabilities of transitioning between high engagement, low engagement, and disengagement during the study. The optimal number of clusters between 2 and 10 was identified visually using the “elbow method” [28]. The elbow method involves plotting the curve of log-likelihood against number of clusters, such that the location of a bend (“elbow”) in the plot is considered to identify the best number of clusters. The clusters were generated by a “blind” algorithmic process. Therefore, to assign names to the clusters, the engagement patterns of a random selection of users within each cluster were inspected.

Comparisons were then made between the clusters regarding duration of study engagement, defined as (1) the median number of days “in study” (defined as the number of days from first to last symptoms report) and (2) the median number of days of data entry (defined as a day when any symptoms were reported). Data completion was compared between the clusters, defined as (1) the total number of segments reported, (2) the total number of complete motifs, (3) the proportion of days in the study (days between enrollment and July 20, 2016) on which complete motifs were reported (days of data entry/total days in study), and (4) the proportion of days of data entry on which complete motifs were reported. Baseline data were then compared between the clusters, with data presented as median and interquartile range (IQR), or proportion and 95% CI where appropriate. Due to the initial configuration of the app, data regarding the mobile-phone platform used by participants are not available for all participants, and we are unable to compare or draw conclusions about how app use differs between Apple and Android platforms.

## Results

Of 7972 participants enrolled in the study between January 20, 2016 and February 29, 2016, 6370 (79.90%) were eligible for the analysis in this paper (Table 1). Reasons for failing eligibility included no baseline data (n=802), age indeterminate (n=308) and age <17 (n=3). A further 489 participants had downloaded the app but never reported symptoms. Those who installed the app but did not prospectively record symptoms did not differ from those who recorded symptoms based on age (median 51; IQR 41-61 vs 49; IQR 41-59) or strength of belief in the association between the weather and pain (median 7; IQR 5-9 vs 7; IQR 6-9). However, a larger proportion were male (30.3%, 95% CI 26.2-34.4 vs 19.5, 18.5-20.5).

Eligible participants were 80.51% (5129/6370) female, with a mean age of 49 years. The majority of those included in the analysis reported pain at more than one site (73.39%, 4675/6370). A further 16.62% (1059/6370) reported pain “all over” and 9.49% (605/6370) reported pain at a single site. The most common diagnosis was arthritis (40.29% type unspecified [2567/6370], 19.12% rheumatoid arthritis [1218/6370]), followed by fibromyalgia/chronic widespread pain (23.75% , 1513/6370) and “other pain diagnosis” (22.64%, 1442/6370). Beliefs about the existence of a relationship between the weather and pain were strong, with a median belief score of 7 (IQR: 6-9). Participants most commonly believed that pain was affected by the damp/rain (74.43% , 4741/6370) and the cold (68.67%, 4374/6370) but least commonly believed that hot weather affected pain (14.76%, 940/6370).

**Table 1:** Baseline characteristics of participants eligible for analysis.

Characteristics			All eligible participants, n (% or SD or IQR^a^) (n=6370)
**Demographics**			
	Female		5129 (80.52%)
	Mean age in years		49.2 (12.9)
**Pain condition**			
	**Site of pain**		
		Single	605 (9.50%)
		Multisite	4675 (73. 39%)
		All over pain	1059 (16.62%)
		Missing	31 (0.49%)
**Diagnosis of conditions**			
	Rheumatoid arthritis		1218 (19.12%)
	Ankylosing spondylitis/spondyloarthropathy		576 (9.04%)
	Gout		231 (3.63%)
	Arthritis (type not specified)		2567 (40.29%)
	Fibromyalgia/chronic widespread pain		1513 (23.79%)
	Chronic headache		462 (7.25%)
	Neuropathic		821 (12.89%)
	Other		1442 (22.64%)
**Medications used at baseline**			
	**Analgesics**		
		None	619 (9.72%)
		Paracetamol	3154 (49.51%)
		Nonsteroidal anti-inflammatory drugs	3694 (57.99%)
		Simple analgesics	1937 (30.41%)
		Weak opiates	1902 (29.86%)
		Strong opiates	782 (12.28%)
		Neuropathic pain medication	1297 (20.36%)
		Other pain medications	717 (11.26%)
	**Disease modifying treatment**		
		None	4407 (69.18%)
		Steroids	480 (7.54%)
		Synthetic DMARDs^b^	1282 (20.13%)
		Biologic DMARDs	560 (8.79%)
		Other DMARDs	406 (6.37%)
**Beliefs**			
	Median strength of belief in the association between weather and pain		7 (6-9)
	**Weather conditions that participants think most affect their pain**		
		Damp or rain	4741 (74.43%)
		Cold	4374 (68.67%)
		Hot	940 (14.76%)
		Changes in barometric pressure	1945 (30.53%)
		Changes in temperature	1967 (30.88%)

^a^IQR: interquartile range.

^b^DMARDs: disease-modifying antirheumatic drugs.

### Comparison With Other Chronic Pain Populations

Compared with data from the Health Survey for England (2011) [23], a greater proportion of participants in this study were women (80.52%, 5129/6370 compared with 55.61%, 4782/8599 expected). The age bands 35 to 64 years were over-represented in this study (73.11%, 4657/6370 compared with 51.18%, 4401/8599 expected; Table 2), with fewer participants in the extremes of age (<35: 14.65%, 933/6370 compared with 24.7%, 2126/8599 expected; ≥75: 1.19%, 76/6370 compared with 11.13%, 957/8599 expected).

### Identifying Clusters of Engagement

**Figure 3:**
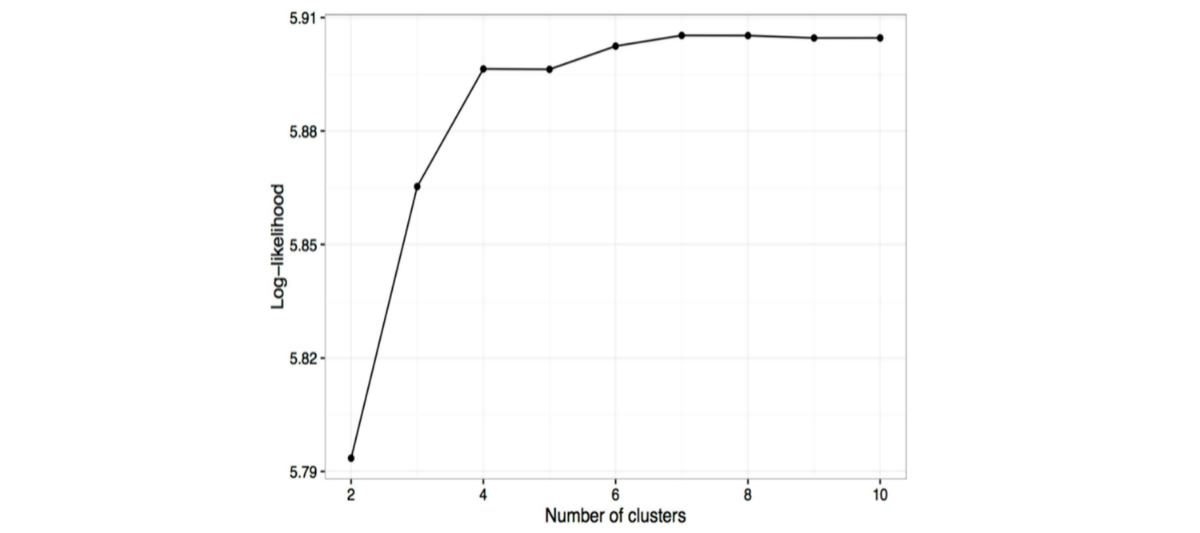
Plot of the log-likelihood of different numbers of clusters in hidden Markov sequences; the elbow indicates the optimal number of clusters which should be accepted.

**Figure 4:**
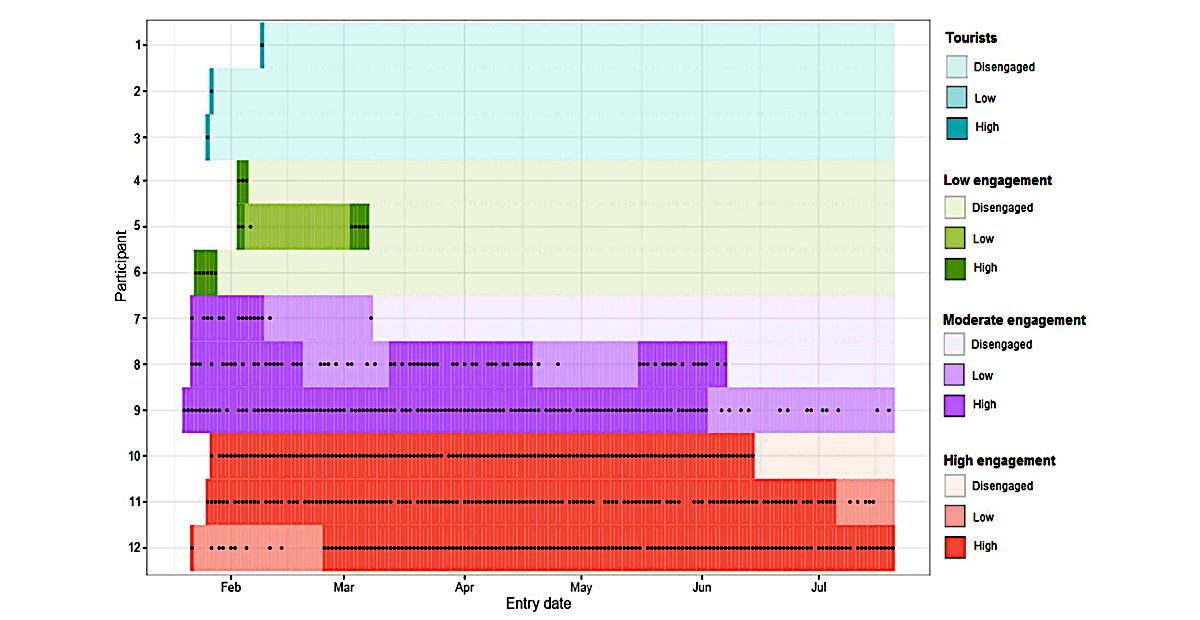
Examples of participants from clusters; High engagement (red), Moderate engagement (purple), Low engagement (green), Tourists (teal).

Following inspection of the log-likelihood plot (Figure 3), a four-cluster solution was retained. The clusters (Figure 4) were allocated names based on the best description of their engagement patterns: high engagement (14%, 865/6370; red), moderate engagement (22%, 1384/6370; purple), low engagement (39%, 2503/6370; green), and tourists (25%, 1618/6370; teal).

The proportion of days on which data were entered and rates of data completion varied substantially between clusters (Table 3).The median days “in study” ranged from 175 days (IQR: 152-177) in the high-engagement cluster to 1 day (IQR: 1-1) in the tourist cluster. Participants in the moderate-engagement cluster stayed in the study 10 times longer than those in the low-engagement cluster (88 days, 42-163 vs 8 days, 4-16).

Those in the high-engagement cluster provided data on most days throughout follow-up (Figure 4). The high-engagement cluster reported complete motifs on 89.13% (106,360/119,332) of the days that they provided data, and the moderate-engagement cluster provided complete motifs on 87.5% (67,704/77,368) of all data-entry days. Rates of completion were slightly lower in the other clusters, with the low-engagement cluster and tourists recording complete motifs on 82.88% (13,415/16,186) and 64.85% (1947/2848) of the days on which any data were reported, respectively (Table 3).

**Table 2:** Comparison of the sex and age distribution of persons with chronic pain from the Health Survey for England (2011) and participants recruited to Cloudy with a Chance of Pain.

Population	Sex		Age (in bands), years					
	Male, n (%)	Female, n (%)	16-34, n (%)	35-44, n (%)	45-54, n (%)	55-64, n (%)	65-74, n (%)	75+, n (%)
Health Survey for England (2011)	3817 (44.39)	4782 (55.61)	2126 (24.72)	1512 (17.58)	1490 (17.33)	1399 (16.27)	1115 (12.97)	957 (11.13)
Cloudy with a Chance of Pain	1231 (19.48)	5129 (80.52)	933 (14.65)	1280 (20.09)	1840 (28.89)	1537 (24.13)	704 (11.05)	76 (1.19)

**Table 3:** Data provided by 6370 Cloudy with a Chance of Pain participants clustered by levels of engagement.

Data	High	Moderate	Low	Tourist
Participants in cluster, n (%)	865 (13.6)	1384 (21.7)	2503 (39.3)	1618 (25.4)
Total number of segments reported	1,233,685	799,872	171,545	26,344
Total number of complete motifs	106,360	67,704	13,415	1847
Total number of days in study	151,187	240,841	435,678	279,755
Total number of days of data entry	119,332	77,368	16,186	2848
Median number of days in study^a^	175 (152-177)	88 (42-163)	8 (4-16)	1 (1-1)
Median number of days of data entry^b^ (IQR^c^)	149 (124-163)	44 (27-80.5)	4 (3-9)	1 (1-1)
Proportion (%) of days in study on which complete motifs were reported	(70.16)	(28.11)	(3.08)	(0.66)
Proportion (%) of days of data entry on which complete motifs were reported	(89.13)	(87.51)	(82.88)	(64.85)

^a^Days between first and final symptom report.

^b^Data entry: any symptom reported.

^c^IQR: interquartile range.

### Between-Cluster Differences

Higher engagement was associated with increased age, with a difference of more than 5 years between the median age of those who were in the low-engagement (47, IQR: 39-57), or tourist clusters (49, IQR: 40-58), and those who were in the high-engagement cluster (median 56 years, IQR: 47-63). A substantially lower proportion of those in the tourist cluster were women (76.27%, 1234/1618; 95% CI 74.2-78.3) than any other cluster (high engagement: 82.31%, 712/865; 95% CI 79.6-84.7; moderate engagement: 84.10%, 1164/1384; 95% CI 82.1-85.9; low engagement: 80.66%, 2019/2503; 95% CI 19.1-82.2).

There were no differences between clusters with respect to the site of pain or in the prevalence of rheumatic disease diagnoses (eg, rheumatoid arthritis, fibromyalgia). The proportion of people in the tourist cluster (17.74%, 287/1618; 95% CI 15.88-19.60) who reported “other” pain conditions was also lower than in the high-engagement (23.70%, 205/865; 95% CI 20.87-26.55), moderate-engagement (24.49%, 339/1384; 95% CI 22.22-26.76), and low-engagement (24.41%, 611/2503; 95% CI 22.73-26.09) groups.

**Table 4:** Characteristics of the 6370 Cloudy with a Chance of Pain participants clustered by levels of engagement.

Data				High (n=865)		Moderate (n=1384)			Low (n=2503)			Tourist (n=1618)		
				n (% or IQR^a^)	95% CI		n (% or IQR)	95% CI		n (% or IQR)	95% CI		n (% or IQR)	95% CI
**Demographics**														
	Female			712 (82.31%)	79.77-84.85		1164 (84.10%)	82.17- 86.03		2019 (80.66%)	79.11-82.21		1234 (76.27%)	74.20-78.34
	Median age in years			56 (47-63)			50 (41-59)			47 (39-57)			49 (40-58)	
**Pain condition**														
	**Site of pain**													
		Single		71 (8.21%)	6.38-10.04		121 (8.74%)	7.25-10.23		226 (9.03%)	7.91-10.15		187 (11.56%)	10.00-13.12
		Multisite		668 (77.23%)	74.44-80.02		1026 (74.13%)	71.82-76.44		1809 (72.27%)	70.52-74.02		1172 (72.44%)	70.26-74.62
		All over pain		121 (13.99%)	11.68-16.30		230 (16.62%)	14.66-18.58		462 (18.46%)	16.94-19.98		246 (15.20%)	13.45-16.95
		Missing		5 (0.58%)	0.07-1.09		7 (0.51%)	0.13-0.89		6 (0.24%)	0.05-0.43		13 (0.80%)	0.37-1.23
**Diagnosis of conditions**														
	Rheumatoid arthritis			176 (20.35%)	17.67-23.03		271 (19.58%)	17.49-21.67		473 (18.90%)	17.37-20.43		298 (18.42%)	16.53-20.31
	Ankylosing spondylitis/spondyloarthropathy			70 (8.09%)	6.27-9.91		130 (9.39%)	7.785-10.93		230 (9.19%)	8.06-10.32		146 (9.02%)	7.62-10.42
	Gout			29 (3.35%)	2.15-4.55		55 (3.97%)	2.94-5.00		80 (3.20%)	2.51-3.89		67 (4.14%)	3.17-5.11
	Arthritis (unspecified)			394 (45.55%)	42.23-48.87		556 (40.17%)	37.59-42.75		958 (38.27%)	36.37-40.17		659 (40.73%)	38.34-43.12
	Fibromyalgia/chronic widespread pain			188 (21.73%)	18.98-24.48		336 (24.28%)	22.02-26.54		634 (25.33%)	23.63-27.03		355 (21.94%)	19.92-23.96
	Chronic headache			43 (4.97%)	3.52-6.42		100 (7.23%)	5.87-8.59		209 (8.35%)	7.27-9.43		110 (6.80%)	5.57-8.03
	Neuropathic			112 (12.95%)	10.71-15.19		182 (13.15%)	11.37-14.93		337 (13.46%)	12.12-14.80		190 (11.74%)	10.17-13.31
	Other			205 (23.70%)	20.87-26.53		339 (24.49%)	22.22-26.76		611 (24.41%)	22.73-26.09		287 (17.74%)	15.88-19.60
**Medications used at baseline**														
	**Analgesics**													
		None		81 (9.36%)	7.42-11.30		115 (8.31%)	6.86-9.76		236 (9.43%)	8.29-10.57		187 (11.56%)	10.00-13.12
		Paracetamol		454 (52.49%)	49.16-55.82		707 (51.08%)	48.45-53.71		1241 (49.58%)	47.62-51.54		752 (46.48%)	44.05-48.91
		Nonsteroidal anti-inflammatory drugs		498 (57.57%)	54.28-60.86		833 (60.19%)	57.61-62.77		1470 (58.73%)	56.80-60.66		893 (55.19%)	52.77-57.61
		Simple analgesics		254 (29.36%)	26.33-32.39		406 (29.34%)	26.94-31.74		773 (30.88%)	29.07-32.69		504 (31.15%)	28.89-33.41
		Weak opiates		253 (29.25%)	26.22-32.28		426 (30.78%)	28.35-33.21		773 (30.88%)	29.07-32.69		450 (27.81%)	25.63-29.99
		Strong opiates		82 (9.48%)	7.53-11.43		154 (11.13%)	9.47-12.79		356 (14.22%)	12.85-15. 59		190 (11.74%)	10.17-13.31
		Neuropathic pain medication		167 (19.31%)	16.68-21.94		278 (20.09%)	17.98-22.20		538 (21.49%)	19.88-23.10		314 (19.41%)	17.48-21.34
		Other pain medications		106 (12.25%)	10.07-14.43		188 (13.58%)	11.78-15.38		270 (10.79%)	9.57-12.01		153 (9.46%)	8.03-10.89
	Steroids			57 (6.59%)	4.94-8.24		96 (6.94%)	5.60-8.28		202 (8.07%)	7.00-9.14		125 (7.73%)	6.43-9.03
	**DMARDs^b^**													
		None		591 (68.32%)	65.22-71.42		974 (70.38%)	67.97-72.79		1690 (67.52%)	65.69-69.35		1152 (71.20%)	68.99-73.41
		Synthetic DMARDs		192 (22.20%)	19.43-24.97		292 (21.10%)	18.95-23.25		529 (21.13%)	19.53-22.73		269 (16.63%)	14.82-18.44
		Biologic DMARDs		80 (9.25%)	7.32-11.18		121 (8.74%)	7.25-10.23		226 (9.03%)	7.91-10.15		133 (8.22%)	6.88-9.56
		Other DMARDs		58 (6.71%)	5.04-8.38		73 (5.27%)	4.09-6.45		156 (6.23%)	5.28-7.18		119 (7.35%)	6.08-8.62
**Beliefs**														
Median strength of belief in the association between weather and pain				7 (6-9)			7 (6-9)			7 (6-9)			7 (5-9)	
	**Weather condition(s) that participants think most affect their pain**													
		Damp or rain		647 (74.80%)	71.91-77.69		1030 (74.42%)	72.12-76.72		1883 (75.23%)	73.54-76.92		1181 (72.99%)	70.83-75.15
		Cold		539 (62.31%)	59.08-65.54		931 (67.27%)	64.80-70.4		1799 (71.87%)	70.11-73.63		1105 (68.29%)	66.02-70.56
		Hot		117 (13.53%)	11.25-15.81		210 (15.17%)	13.28-17.06		383 (15.30%)	13.89-16.71		230 (14.22%)	12.52-15.92
		Changes in barometric pressure		307 (35.49%)	32.30-38.68		455 (32.88%)	30.41-35.35		714 (28.53%)	26.76-30.30		469 (28.99%)	26.78-31.20
		Changes in temperature		238 (27.51%)	24.53-30.49		422 (30.49%)	28.06-32.92		797 (31.84%)	30.01-33.67		510 (31.52%)	29.26-33.78

^a^IQR: interquartile range.

^b^DMARDs: disease-modifying antirheumatic drugs.

No differences were observed between the clusters regarding the use of analgesics and steroids. Only the use of synthetic DMARDs differed substantially between the clusters, with less of those in the tourist cluster (16.63%, 269/1618; 95% CI 14.82-18.44) reporting taking the medication than those in the other engagement clusters (high engagement: 22.20%, 192/865; 95% CI 19.43-24.97; moderate engagement: 21.10%, 292/1384; 95% CI 18.95-23.25; low engagement: 21.13%, 529/2503; 95% CI 19.53-22.73). Comparable proportions were using biologic or other DMARDs.

There were no differences in the strength of belief that the weather affected pain, but fewer of those in the high-engagement cluster believed the cold affected their pain (62.31%, 539/865; 95% CI 59.08-65.54) when compared with those in the low-engagement and tourist clusters (71.87%,1799/2503; 95% CI 70.11-73.63 and 68.29%, 1105/1618; 95% CI 66.02-70.56, respectively). Conversely, more of those who were highly engaged (35.49%, 307/865; 95% CI 32.30-38.68) believed that changes in barometric pressure were associated with pain that those in the low-engagement and tourist clusters (28.35%, 714/2503; 95% CI 26.76-30.30 and 28.99%, 469/1618; 95% CI 26.78-31.20, respectively). There were no observed differences in the proportion of participants who believed their pain is associated with damp or rain, heat, or changes in temperature (Table 4).

## Discussion

### Principal Findings

Cloudy with a Chance of Pain is the first mHealth study to demonstrate successful and rapid mass recruitment of a largely representative sample of highly engaged participants. Among our sample, patterns of ongoing engagement showed that around 1 in 7 participants provided data on most days in the first 6 months, completing full data entry on 89% of those days.

A major strength of Cloudy with a Chance of Pain is the rapid mass recruitment of eligible participants. Our study benefitted from wide promotion by the UK national media at the time of the study launch, which emphasizes the power of national media to promote. Indeed, as a result of coverage including, among others, the BBC2 television show *Trust Me I’m a Doctor* on January 20, 2016 and BBC *Breakfast* on January 26, 2016, 90% of participants enrolled in the study by July 20 were recruited within 1 month of the study launch.

Furthermore, ongoing engagement within Cloudy with a Chance of Pain was high. More than 30% of participants were in the high-engagement or moderate-engagement cluster, entering data on at least half of days throughout the 6 months. In comparison, fewer than 25% of participants in Apple’s ResearchKit studies were active by 10 weeks [29], with similar proportions active in a physical-activity study by 42 days [14]. In one of the largest mHealth studies reported to date (mPower study of people with Parkinson disease and healthy controls), less than 10% of enrolled participants completed 5 or more days within the first 6 months of the study [5]. One in 7 participants were in the high-engagement cluster and provided data on most days throughout the 6 months; we are not aware of other mHealth studies that have reported such high levels of ongoing engagement to date.

Previous analyses have used arbitrary definitions that fail to capture the patterns of use through time and may ignore the importance of continuity of data entry [5,14-16]. In contrast, this analysis attempted to account fully for data complexity and made no a priori decisions to define engagement. Thus, this study has improved understanding of the extent to which participants remain engaged over time and provides a promising method for future engagement studies.

Our recruitment strategy enrolled a sample which comprised an under-representation of males and persons at the extremes of age (<35 years and ≥75 years) than would have been expected from the general population data of the Health Survey for England (2011) [23]. Although women are more likely to respond to more traditional population surveys [30-33], we recruited a much higher proportion of women than would have been expected using traditional recruitment methods. A possible explanation is that women recruited to this study more commonly viewed the television programs or may have perceived the potential for additional benefits for participation. However, we also note that self-selection likely accounts for the observed differences in our population. For example, not only are women known to use social media [34] and health apps more than men [35], but they also use digital content differently [36,37].

Therefore, future mHealth studies may benefit from the use of supplementary and targeted recruitment strategies used by other digital health interventions [38] in which it would be possible to oversample men, such as the use of health professionals, friends, and families, or work-based campaigns, as well as outreach programs designed to access hard-to-reach groups. Although similar methods could also promote the recruitment of younger adults, other opportunities to promote participation among this group include the use of social networks, community components, and app gamification [39-41].

Nevertheless, the internal validity of the study results (ie, the relationship between the weather and pain within our sample) is unlikely to be influenced by the excess of women entering the study, as there is no reason to suspect the relationship differs by sex. Analysis of the relationship between the weather and pain, and whether the relationship differs between the sexes, is underway and will be reported separately.

The impact on external validity (ie, the generalizability) is unclear, as people with a particular belief may have been more inclined to participate (which may, in turn, differ by sex). That said, our findings about beliefs align with prior research that suggests that as many as 92% of patients with arthritis believe in an association between weather and pain [42].

The reasons for the unprecedented rates of engagement observed in this study are worth exploring, particularly as we sought to collect a large amount of daily data, and this burden on the participants might well have been expected to result in a higher loss to follow-up through time and over such a long period. This study found that older participants were more likely to remain engaged in the study. One possible explanation for this is that older persons are less likely to use smartphone apps [1] and therefore may be less likely to experience “app fatigue” than younger participants. They may also feel a greater responsibility to complete the ongoing data entry once registered or have more time to give to the study. Furthermore, functionalities such as geolocation consume battery power, which may have a greater impact on younger persons, who use their smartphones for a greater number of varied tasks, than on older persons [1]. We note, however, that reasons for declining engagement are likely numerous.

Earlier studies have sought to examine possible mechanisms of engagement, including the complexity of tasks [3], the time of day data are entered [43], and various functionality features such as reminders, interactivity, tailored content, and delivery of feedback [14,15]. In a feasibility study [22], we reported that key motivators for ongoing engagement were the simple graphical user interface, automated reminders for data entry, a desire to contribute to answering an understandable and engaging research question, and visualization of data. However, limited information was available in this larger study to delineate the motivators of engagement in this population. We also acknowledge that the study did not capture education and income, which would have enabled this study to investigate the potential impact of the digital divide on recruitment and engagement.

### Conclusions

In summary, Cloudy with a Chance of Pain demonstrates a rapid and successful recruitment of a large and engaged sample of people with chronic pain. Although there may be selection bias toward older females in our study, younger men are also less likely to participate in studies using traditional data-collection methods. Thus, our study provides strong evidence to suggest that smartphones could provide a viable alternative to traditional data collection methods, particularly for collecting daily data over long periods.

## Appendix

Multimedia Appendix 1 Charity and patient partner organisations who facilitated participant recruitment.

Multimedia Appendix 2 Hidden Markov model.

## Abbreviations

DMARDs: disease-modifying antirheumatic drugs
GPS: global positioning system
HSE: Health Survey for England
iOS: iPhone operating system
IQR: interquartile range
mHealth: mobile health
NRS: numerical rating scale
NSAIDs: nonsteroidal anti-inflammatory drugs
OA: osteoarthritis
