# Supplementary material for: Recruitment and Ongoing Engagement in a UK Smartphone Study Examining the Association Between Weather and Pain: Cohort Study
Source: JMIR Mhealth Uhealth. 2017 Nov 1;5(11):e168. doi: 10.2196/mhealth.8162 (PMC5688244; doi:10.2196/mhealth.8162)
Supplement: Multimedia Appendix 1 [file mhealth_v5i11e168_app1.pdf]

Multimedia Appendix 1 - Charity and patient partner organisations who facilitated participant recruitment

Action for M.E

Arthritis Action

Arthritis Research UK

Burning Nights

CRPS UK

Diabetes UK

Ehlers Danlos UK (EDS UK)

Erythromelalgia Warriors (EM Warriors)

Fibromyalgia Action UK

Lupus UK

Migraine Action

The Migraine Trust

National Ankylosing Spondylitis Society (NASS)

The National Rheumatoid Arthritis Society

Pain Concern

Postural Orthostatic Tachycardia Syndrome (PoTS UK)

PsAZZ – Psoriatic Arthritis Support Group

Scleroderma & Raynaud’s UK (SRUK)

UK Gout Society
