# Supplementary material for: Recruitment and Ongoing Engagement in a UK Smartphone Study Examining the Association Between Weather and Pain: Cohort Study
Source: JMIR Mhealth Uhealth. 2017 Nov 1;5(11):e168. doi: 10.2196/mhealth.8162 (PMC5688244; doi:10.2196/mhealth.8162)
Supplement: Multimedia Appendix 2 [file mhealth_v5i11e168_app2.pdf]

Multimedia Appendix 2 - Hidden Markov model

A simple way to model participants' entry or non-entry of data is with a first-order Markov model. That is: given that I submitted data today, what is the probability that I will enter data tomorrow? What is my probability of entering data tomorrow if I did not submit data today? One potential problem with such an approach is that there is no distinction made between days of non-data-entry in the middle of the period (which we might term 'inactivity') and days after the participants' final recorded data point, at which time we can say the user has dropped out. Moreover, having missed a day here or there should not necessarily increase a participants' estimated likelihood of dropping out.

A more advanced model for user behaviour is a first-order hidden Markov model. This involves 'hidden' or 'latent' states, so that we no longer consider transitions between data entry and non-entry, but transitions between the (unobservable) states. For more information on hidden Markov models, try Barber, D. (2012). Bayesian Reasoning and Machine Learning, chapter 23. The proposed model incorporates three latent states.

high engagement high probability of submitting data, low probability of changing state

low engagement low probability of submitting data, low probability of changing state

disengaged zero probability of submitting data, zero probability of changing state

Here, "disengagement" is called an 'absorbing' state (i.e. one from which you cannot return), because once a participant drops out, they cannot re-enter the study. Thus a participant can experience low engagement in the middle of the study but only becomes disengaged if no future data are submitted.

To fit the hidden Markov model, first we need to obtain a binary sequence corresponding to submission and non-submission of data, running from the date of each participants' first submission until the end of the study period on 20 July 2016.

Clustering by engagement

Typical ways of looking at "engagement" in epidemiological studies categorise participants according to the data submitted as a proportion of the total data they could have submitted. For example, if participants submitted data on more than x% of days that they were enrolled in the study, they might be considered "highly engaged", where x% is some threshold (say 80%), and considered "lowly engaged" otherwise.

As this does not capture all the different ways in which participants might behave, and the threshold can be somewhat arbitrary, we can consider clustering them according to their levels of engagement as inferred in the previous section.

To do this, we fit a Markov mixture model to describe the sequences of high and low engagement and disengagement learned from the participants. The mixture is computed using an expectation maximisation (EM) algorithm. As an EM algorithm is liable to converge to local optima, we initiate the algorithm several times and choose the result with the highest log-likelihood. From this output,

participants are assigned to clusters according to the respective posterior probabilities of the mixture components.
